# A strategy for building and using a human reference pangenome

**DOI:** 10.12688/f1000research.19630.2

**Published:** 2021-07-29

**Authors:** Bastien Llamas, Giuseppe Narzisi, Valerie Schneider, Peter A. Audano, Evan Biederstedt, Lon Blauvelt, Peter Bradbury, Xian Chang, Chen-Shan Chin, Arkarachai Fungtammasan, Wayne E. Clarke, Alan Cleary, Jana Ebler, Jordan Eizenga, Jonas A. Sibbesen, Charles J. Markello, Erik Garrison, Shilpa Garg, Glenn Hickey, Gerard R. Lazo, Michael F. Lin, Medhat Mahmoud, Tobias Marschall, Ilia Minkin, Jean Monlong, Rajeeva L. Musunuri, Sagayamary Sagayaradj, Adam M. Novak, Mikko Rautiainen, Allison Regier, Fritz J. Sedlazeck, Jouni Siren, Yassine Souilmi, Justin Wagner, Travis Wrightsman, Toshiyuki T. Yokoyama, Qiandong Zeng, Justin M. Zook, Benedict Paten, Ben Busby

**Affiliations:** 1 Australian Centre for Ancient DNA, School of Biological Sciences, Environment Institute, The University of Adelaide, Adelaide, South Australia, 5005, Australia; 2 New York Genome Center, New York, NY, 10013, USA; 3 National Center for Biotechnology Information, National Library of Medicine, National Institutes of Health, Bethesda, MD, 20894, USA; 4 Department of Genome Sciences, University of Washington School of Medicine, Seattle, WA, 98195, USA; 5 Kravis Center for Molecular Oncology, Memorial Sloan Kettering Cancer Center, New York, NY, 10065, USA; 6 Department of Biomedical Informatics, Harvard Medical School, Boston, MA, 02215, USA; 7 Genomics Institute, University of California, Santa Cruz, Santa Cruz, CA, 95064, USA; 8 Robert W. Holley Center, USDA-ARS, Ithaca, NY, 14853, USA; 9 DNAnexus, Mountain View, CA, 94040, USA; 10 National Center for Genome Resources 87505, Santa Fe, NM, 87505, USA; 11 Max Planck Institute for Informatics, Saarbrücken, Germany; 12 Department of Genetics, Harvard Medical School, Boston, MA, 02115, USA; 13 Western Regional Research Center, USDA-ARS, Albany, CA, 94710-1105, USA; 14 mlin.net LLC, San Jose, CA, 95113, USA; 15 Human Genome Sequencing Center, Department of Molecular and Human Genetics, Baylor College of Medicine, Houston TX, TX, 77030, USA; 16 Department of Computer Science and Engineering, The Pennsylvania State University, University Park, PA, 16802, USA; 17 Genome Center, University of California, Davis, Davis, CA, USA; 18 BASF, West Sacramento, CA, USA; 19 McDonnell Genome Institute, Washington University in St Louis, St Louis, MO, 63108, USA; 20 Material Measurement Laboratory, National Institute of Standards and Technology, Gaithersburg, MD, 20899, USA; 21 Section of Plant Breeding and Genetics, Cornell University, Ithaca, NY, 14853, USA; 22 Department of Computational Biology and Medical Sciences, Graduate School of Frontier Sciences, The University of Tokyo, Chiba, Japan; 23 Laboratory Corporation of America Holdings, Westborough, MA, 01581, USA

**Keywords:** Hackathon, Pangenome, Graph Genome, RNAseq, Structural Variant

## Abstract

In March 2019, 45 scientists and software engineers from around the world converged at the University of California, Santa Cruz for the first pangenomics codeathon. The purpose of the meeting was to propose technical specifications and standards for a usable human pangenome as well as to build relevant tools for genome graph infrastructures. During the meeting, the group held several intense and productive discussions covering a diverse set of topics, including advantages of graph genomes over a linear reference representation, design of new methods that can leverage graph-based data structures, and novel visualization and annotation approaches for pangenomes. Additionally, the participants self-organized themselves into teams that worked intensely over a three-day period to build a set of pipelines and tools for specific pangenomic applications. A summary of the questions raised and the tools developed are reported in this manuscript.

## Introduction

### What is pangenomics?

The current human reference genome, GRCh38 ( Schneider *et al.*, 2017), derives from a draft sequence that was constructed from a handful of individuals ( Lander *et al.*, 2001) likely of African and European ancestries ( Reich *et al.*, 2009). Today, GRCh38 captures a limited amount of additional genetic variation by providing alternative sequence representations (“alt loci”) for complex or highly variable regions, such as the SMA and MAPT loci on chromosomes 5 and 17, respectively ( Schneider *et al.*, 2017), whose sequence is derived from additional DNA samples. However, analyses of other individual human genome assemblies from Europeans ( Ameur *et al.*, 2018; Audano *et al.*, 2019; Kidd *et al.*, 2010; Levy *et al.*, 2007; Wheeler *et al.*, 2008), East Asians ( Audano *et al.*, 2019; Kidd *et al.*, 2010; Li *et al.*, 2010; Seo *et al.*, 2016; Shi *et al.*, 2016), South Asians ( Audano *et al.*, 2019; Kitzman *et al.*, 2011), Amerindians ( Audano *et al.*, 2019) and Africans ( Audano *et al.*, 2019; Kidd *et al.*, 2010; Li *et al.*, 2010; Sherman *et al.*, 2019) have still revealed a substantial amount of genomic information not represented in the reference assembly. Indeed, large re-sequencing projects showed an extensive human genetic diversity, even within the genomic content captured in the reference sequence ( Bycroft *et al.*, 2018; 1000 Genomes Project Consortium *et al.*, 2010; Mallick *et al.*, 2016). Although GRCh38 is the most complete human reference to date, it is not clear how to construct a linear reference that can capture diversity and address population biases that impact analysis ( Brandt *et al.*, 2015; Degner *et al.*, 2009).

### Why is a pangenome representation superior to the current human reference assembly model?

The diploid structure of human DNA is not currently represented in the current reference model, which is instead an arbitrary linear combination of different haplotypes (i.e., a mosaic) from multiple individuals. A human “pangenome” is a representation of all genomic variation observed in human populations ( Computational Pan-Genomics Consortium, 2018). In this context, a pangenome is a more comprehensive representation of genetic diversity than an individual diploid genome or a reference comprised of linear chromosomes built from multiple individuals, such as GRCh38. By extension, pangenomics encompasses approaches that utilize a pangenome reference. Pangenomics is designed to address the limitations of current standards, such as reference bias during the identification of genomic variants, population stratification and admixture, or ancestry-specific functional variants—among others, which impact evolutionary, agricultural and health genetics research. For example, reference bias in the sequence alignment to GRCh38 (excluding its alt loci) reduces our ability to correctly genotype regions that are likely to significantly diverge from the reference chromosome representations—e.g. immune regions such as the major histocompatibility complex (MHC) and killer cell immunoglobulin-like receptors (KIR), and the CYP2D6-8 loci involved in drug metabolism ( Dilthey *et al.*, 2015). Alignment around indels becomes more challenging as their size increases with soft-clipping being preferred over split-read alignment ( Garrison *et al.*, 2018; Narzisi *et al.*, 2014). Variants cannot be identified within regions completely missing from the reference sequence, many of which have been recently identified to be common across individuals ( Taliun *et al.*, 2019). Although bias and missing sequence may still persist in a pangenome, their effects should be substantially less, and may even be ameliorated by adding new content to the framework. In addition to these issues with the current reference, several studies using long reads have reported an average of ~20,000 structural variants (SV) per human genome, most of which fall within repetitive elements and segmental duplications (HGSVC) ( Audano *et al.*, 2019; Chaisson *et al.*, 2015). Many of these SVs intersect genes and regulatory elements, harbor transposable elements, and affect gene expression ( Audano *et al.*, 2019; Chiang *et al.*, 2017). Although they are largely inaccessible to short-read sequence with current methods, these variants can be more easily re-identified using a pangenome ( Chen *et al.*, 2019; Hickey *et al.*, 2020). Complex loci that harbor multiple repeats are also quite challenging to detect and genotype by aligning reads to a linear reference. Important disease-linked repeats, such as the CAG repeat in the HTT gene that causes HD and the CAG repeat in *ATXN8* that causes Spinocerebellar ataxia type 8 (SCA8), are both flanked by other polymorphic repeats making them particularly difficult to accurately genotype. Sequence graphs offer again a general and a more flexible approach to handle these complex loci ( Dolzhenko *et al.*, 2019).

### What is a haplotype?

The International HapMap Consortium defines a haplotype as “a particular combination of alleles along a chromosome” ( International HapMap Consortium, 2005). A diploid individual has two haplotypes for any given genomic sequence—up to the complete genome itself—since it inherits a set of homologous chromosomes from each parent ( Crawford & Nickerson, 2005). At the population level, there may be more than two haplotypes for any given sequence. The definition of haplotype will vary in the scientific literature depending on discipline-specific questions and applications ( Hoehe, 2003). For evolutionary and population geneticists, haplotype may be short for haplotype block, which is a group of alleles that are inherited together across multiple generations and results from recombination and selection; the arrangement and length of haplotype blocks will inform about past population history ( Wang *et al.*, 2002). For medical geneticists, haplotype may represent a functional haplotype at the gene level, i.e. genetic markers linked to a disease-associated allele in so-called linkage disequilibrium, or LD ( Slatkin, 2008). For livestock and crop breeders, a haplotype may be the minimal genomic region that influences a trait of interest ( Hayes, *et al.*, 2013; Qian *et al.*, 2017). Whatever the definition of a haplotype, haplotypic information can simultaneously provide clues about population history and disease or trait association ( Martin *et al.*, 2018).

### Why is phasing important?

Today’s widespread use of short-read sequencing provides easy access to genotypes but does not necessarily directly inform about the parental origin of each allele. However, the real power of haplotypes resides in phasing, which is the assignment of a given combination of alleles to each homologous chromosome ( Browning & Browning, 2011). Beyond the methodological challenge of phasing genomes ( Choi *et al.*, 2018), the two haploid sequences in a diploid genome cannot be captured simultaneously in one linear sequence. However, a genome graph representation of a pangenome provides a spatial framework to embed multiple haplotypes at once and preserve phasing information ( Paten *et al.*, 2017). This property of graph representations of a genome is critical. At the gene scale, phasing information can be used to recognize compound heterozygosity, whereby the two homologous copies of a gene are each affected by a distinct recessive mutation ( Snyder *et al.*, 2015). Phenotype prediction depends heavily on the ability to distinguish point mutations or deletions between chromosomes ( Cirulli & Goldstein, 2010; Tewhey *et al.*, 2011), making the retention of phasing information fundamental for the interpretation of results in a personalised medicine setting. Other applications of phasing include the inference of past population demographic history by looking at the distribution and size of haplotype blocks along chromosomes ( Schiffels & Durbin, 2014). Variant imputation also depends heavily on the availability of phasing information and becomes a key approach in large cohort studies with missing genotypes ( Das *et al.*, 2018). Finally, the sequencing of fetal cell-free DNA in maternal plasma is a very promising way to study fetal genomes in a non-invasive manner. However, it is first essential to phase haplotypes from at least one of the parents ( Fan *et al.*, 2012; Kitzman *et al.*, 2012).

## Methods

Here we describe the data sets and graph construction techniques used during the codeathon, as well as the pipelines and software that were developed.

## Implementation

### Graph coordinates system

To establish protocols to build pangenomic graphs from chromosome-level and ultra-long assemblies, we constructed graphs using the human reference genome GRCh38.p13, CHM1 cell-line data, and two primate references: chimpanzee (PanTro PTRv2; Clint; GenBank assembly accession GCA_002880755.3) and Sumatran orangutan (PonAbe3 PABv2; Susie; GenBank assembly accession GCA_002880775.3). Additionally, we built human-only graphs using the human reference genome (GRCh38.p13; GenBank assembly accession GCA_000001405.28) and the Japanese reference genome (JG1; available at https://jmorp.megabank.tohoku.ac.jp/201902/downloads/).

We used three different methods to build the graph and explore the potential limitations and advantages of each method. These methods were chosen as they allow us to explore evolutionary questions, such as ancestral states, large structural variations between groups, and complex gene genealogies. They were are used for their computational tractaibility in the limited time frame of the 3-day hackathon. We first created graphs based on sequences from chromosome 21 from GRCh38 (CM000683.2), Clint the chimpanzee (CM009259.2), Susie the Sumatran orangutan (CM009283.2), and CHM1 (AC244111.3, AC244144.2, AC244518.2, AC245051.3, AC245314.2, AC246819.2, AC255431.1, AC256301.1, AC277730.1, AC277802.1, AC277887.1).

Graph method 1: We used minimap2 (v2.16-r922) ( Li, 2018) with the parameter preset asm5 to do an all-vs-all alignment of the sequences. We then used seqwish (6e4fe705;) to induce a graph in GFAv1 (Graphical Fragment Assembly) format, and converted this to VG format ( Garrison *et al.*, 2018) for further investigation.

Graph method 2: We used Cactus ( Paten *et al.*, 2011), which is designed to build genome graphs of different taxa while accounting for the phylogenetic relationship between the organisms included. The generated Cactus graph in HAL format was converted to VG format using hal2vg for mapping and visualization.

Graph method 3: We used SibeliaZ ( Minkin & Medvedev, n.d.) to build a graph from chr1 of JG1 and GRCh38.

**Figure 1.**
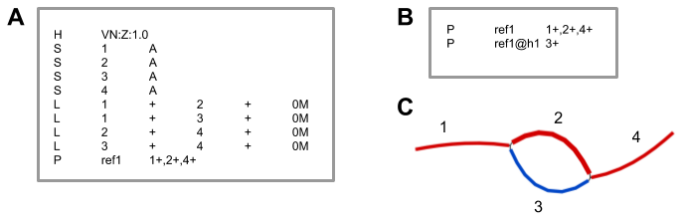
Proposed graph coordinate system to represent multiple haplotypes. **A**) Example of a GFA file ( https://github.com/GFA-spec/GFA-spec) that represents a reference genome and one alternate haplotype. The first line beginning in ‘H’” is the header, with an optional 'VN' SAM-tag version number. Nodes, represented by lines starting with ‘S’, have a name in the second column and a nucleotide sequence in the third column. Edges, represented by lines starting with ‘L’, connect nodes whose sequence appears adjacent to each other in one of the haplotypes. The node names appear in the second and fourth columns, and the orientations appear in the third and fifth columns. The line beginning with ‘P’ is from GFA version 1, and encodes subgraphs and paths. **B**) A path file accompanying the GFA file includes paths for the reference genome and haplotype 1. The haplotype name is in column 2 and the sequence of nodes and their orientations are in column 3. The nucleotide sequence for any haplotype can be resolved by reading out the sequence for each node in the path. **C**) Visualization of **A** using path labels from **B**. The red path represents ref1, while the blue path represents haplotype ref1@h1.

Finally, we designed a prototype of a graph coordinates system based on previously proposed ideas ( Rand *et al.*, 2017) that streamlines the incorporation of new haplotypes into the graph, while preserving a structure that is retro-compatible with the GRCh38 linear reference coordinates ( Figure 1). Such a coordinate system offers a host of advantages, as it allows easier surjection/projection of graph coordinates onto the linear reference coordinates. It also streamlines variant discovery and improves annotation portability.

### A faster, better short-read mapper with hit chaining

**Figure 2.**
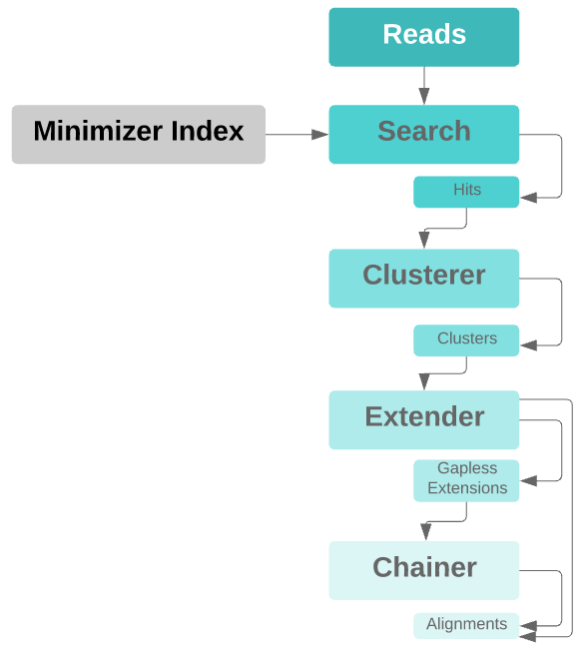
Pipeline diagram of the mapper. Input reads are scanned for minimizers, which are searched against a precomputed minimizer index of the graph reference. Minimizer hits for sufficiently rare minimizers are located in graph space, and the hits for all minimizers are clustered. The clusters are extended gaplessly, with a tolerance for mismatches. If a cluster produces a single full-length gapless extension, it is output as the alignment. Otherwise, partial gapless extensions are chained together by performing alignments of the intervening sequences and graph paths that connect them.

Our work modifies vg ( Garrison *et al.*, 2018; Hickey *et al.*, 2020) to create a fast and efficient read mapper. During the codeathon, we have improved a prototype minimizer-based mapper by adding a faster clustering function to cluster minimizer hits and hit extension logic for handling clusters that have no good full-length gapless alignment ( Figure 2).

The clustering algorithm has been improved by reducing the amount of data copying in the clustering implementation. Alignments may be output from the extender if chaining is not necessary. Additionally, we have devised an improved algorithm for comparing sets of clusters.

We also implement hit chaining which allows us to deal with crossovers and indels. When the extender cannot find a full-length gapless extension of the read alignment to some haplotype with below a threshold number of mismatches, where it previously would leave the read unaligned, it will instead now compute maximal unambiguous-path exact matches between the read and the graph’s embedded haplotypes and feed them to an extension step. The extension step will trace out the haplotype segments that could connect between those matches, perform gapped alignment of the relevant read sequence against each, and take the best for each possible connection. Then the resulting multipath alignment will be linearized into an optimal gapped single-path alignment for the read.

### Pipeline for mapper evaluation on maize graphs

**Figure 3.**
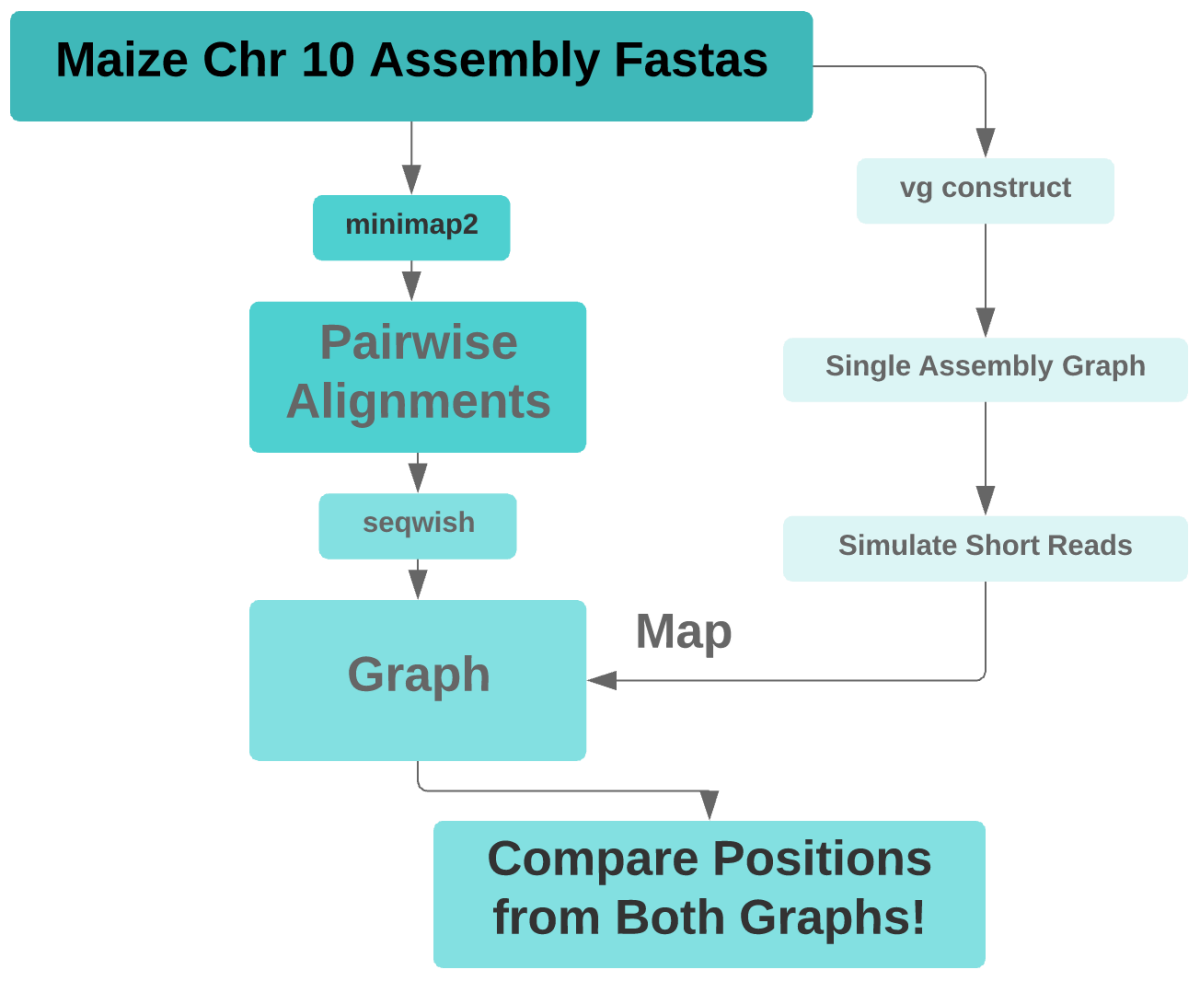
Pipeline diagram for mapper evaluation on *Zea mays* graphs. After constructing graphs with vg construct and with minimap2 and seqwish (Graph method 1), we sought to simulate reads from the vg construct graph, align them to the minimap2/seqwish graph with our faster, better short read mapper with hit chaining, and then to evaluate the mapper’s accuracy based on the simulated reads’ original and realigned positions along corresponding positional paths in the two graphs.

Pangenomics naturally has applications outside of human genomics, and we sought to test how current graph genome methods would apply to genomes more complex in terms of ploidy and variation.tWe also sought to test a plant mode For this, we chose the maize (Zea mays) genome, which is 2.3 Gb in length with 10 chromosomes and contains over 32,000 protein-coding genes ( Schnable *et al*., 2009). A total of 85% of this genome has been estimated to contain transposable elements (TE) ( Schnable *et al*., 2009). Using chr 10, we composed a graph using vg construct and compared it to a graph created with minimap2 for alignment and seqwish (for converted iting to GFA1 format with seqwish (Graph method 1) ( Figure 3).

We could not index the minimap2/seqwish graph for mapping because it contained extremely large snarls, with hundreds of thousands of net graph nodes. One of the indexes we needed to produce, the distance index, which is used for identifying nearby seed hits for clustering, requires doing an all-against-all distance computation on the net graph of each snarl, and that process tried to allocate more memory to hold its result than waswe had provisioned for the hackathonon our machine. We thus aborted the experiment at that step.

We believe that the graph we generated, shown in Supplemental Figure 1 (available as *Extended data*) as an odgi visualization, was pathologically complex and intractable, because we did not remove spurious, short alignments from the minimap2 output. The intractability of this graph precluded further analysis.

### Mapping RNA sequencing data to variant graphs

Using known variants and haplotypes during mapping of RNA sequencing (RNA-seq) data have shown to be important for reducing reference bias and thus improving downstream analyses. Reference bias is known to negatively impact estimation of allele-specific expression ( Degner *et al.*, 2009) and variant-aware mapping is one of the best ways to mitigate this problem ( Castel *et al.*, 2015). Furthermore, it has been shown that inference of gene expression in the highly polymorphic MHC can be improved by using the alternative reference haplotypes during mapping ( Lee *et al.*, 2018). A few variant-aware methods for mapping of RNA-seq reads exist, including GSNAP (Genomic Short-read Nucleotide Alignment Program) ( Wu & Nacu, 2010) and Hisat2 ( Kim *et al.*, n.d.). Hisat2 is similar to vg in that it is also based off of a graph representation.

We wanted to test whether we could also use vg to map RNA-seq reads to a graph containing both known variants, splice-junctions and haplotype-specific transcript paths. We called this a spliced variation graph. We further wanted to show that we could use the reads mapped to the graph to get unbiased estimates of allele-specific transcript expression. The pipeline would serve as a proof of concept for a graph based approach for inferring allele-specific transcript expression when an individual's haplotypes are available, similar to the personal genome approach ( Rozowsky *et al.*, 2011).

### Assessment of mutation rates in and around structural variants using graph genomes

Mutation rates vary across the genome with certain hotspots associated with accessible regions as well as other genomic features. This is also discussed in the presence of gene duplication where in a single copy gene case the mutations are rare due to the selection pressure. However, this selection pressure is reduced when there are two or more copies of the gene, and higher mutation rates are possible for at least one copy of the gene.

To assess the presence of SNPs inside SVs, we constructed a graph genome in vg ( Garrison *et al.*, 2018) to incorporate the SVs found in a recent *Cell* paper ( Audano *et al.*, 2019). This highlights one application where graph genomes might provide improved insight over traditional mapping approaches. To assess this we used SNP calls for HG002, a gold standard in genomics reported to be present based on the Genome In A Bottle (GIAB) consortium ( Zook *et al.*, 2016). We compared the power of vg over short Illumina reads and Pacific Biosciences (PacBio) Circular consensus sequencing (CCS) reads and PacBio continuous long reads (CLR). Subsequently we extended our project to additional samples, focusing on the assessment of mutation rates inside common SVs between the Caucasian and African populations. This revealed changes in mutation rates when looking at tandem duplications between the flanking and the affected regions. It would be interesting to scale this project further for larger cohort samples to assess the mutation rate across multiple samples and ethnicities. This could help understand if SVs are indeed the driver for certain phenotypes, or if the variations within the SVs are more likely to impact the phenotypes.

The code to generalize this analysis for larger cohorts such as the 1000 Genomes Project or Simons Genome Project samples is available on GitHub (See “Data and software availability”).

### Implementing annotations on pangenome graphs

Linear genomes currently rely on genomic intervals as a core formalism for annotation but it is difficult to generalize this formalism to reference graphs. A genomic interval corresponds to a path through a graph. However, if we restrict the annotation to one path in the graph, the alternate alleles in the graph are not included in the annotation. We argue that connected subgraphs are a more appropriate formalism for annotating genome graphs. Using a new core formalism for annotation necessarily means that infrastructure to manipulate it does not yet exist. We need stable and exchangeable representations of the data, software support, and analysis tools to make the formalism useful for practitioners. We have developed a proof-of-concept system for projecting linear reference annotations onto genome graphs and utilizing them in downstream visualizers and analyses. The standard file format, named gGFF, has been defined on GitHub and code to manipulate and use this file format has been included in vg. We also developed a tool for performing utility operations on gGFF files, such as intersection and union.

A common use of annotations is generating gene or transcript-level counts of RNAseq read mappings for differential expression analysis. We have implemented an example RNA-seq quantification pipeline using a graph constructed from GRCh38 ch21 and variants from the 1000 Genomes Project. We converted this to a splice site-aware graph with *vg rna*. The next step would be to map RNA-seq reads to this graph and estimate coverage per base-pair using *vg pack* and gene-level quantification computed using GENCODE 29 annotation.

## Operation

The software should run on most Linux installations. Interested parties are encouraged to clone the GitHub repository and follow the workflow/instructions provided for the individual implementations of the Use cases listed below. Pull requests and contacting the authors is strongly encouraged. 

## Results and use cases

Fundamentally, the motivation behind exploring graph genomes lies in the novel insights we may gain with their applications ( Eizenga *et al*., 2020). There are also regions— -- outside the alternative loci that are defined for GRCh38— -- that cannot easily be reduced to a single linear reference, and telomere-to-telomere de novo assembly of each individual genomes ( Logsdon *et al*., 2021; Miga *et al*., 2020) will likely be implausible on a large scale for the foreseeable future. Graph genomes can be used for inference of extension and phasing from sparse information derived from SNP chips and RNA-seq. They can also be used to infer allele- specific expression on an individual level. Additionally, there is development of methods to represent variation in the clinically important MHC locus, and explore this locus at a population level ( Chin *et al*., 2020; Dilthey, 2021).

Finally, in theory, having clusters of haplotypes within and across populations will allow us to efficiently determine the relationships of proximal and distal phenotype-relevant events.

Taking these points together, a pangenome graph would likely result in a reduction in the “total cost of ownership of genomes”; i.e. people can use information derived from graphs instead of remapping to a linear genome over and over again, expending computer resources needlessly to createing novel .bams/.vcfs files *ad infinitum*.

### Use case: Integrating haplotype information into reference genome with retro-compatibility

Representing haplotype information in reference genomes is beneficial in increasing mappability and reducing bias. The major concerns for representing haplotypes in the existing reference genome are the alteration of coordinates, redundant representation, and ambiguous sequence inference. Our proposed notation tackles these issues with the following design philosophies:

1- The haplotype contigs are coordinated and defined as an add-on outside the extant reference genome coordinates. This allows the set of haplotype contigs to be updated separately, and the inclusion of haplotype sequence does not alter the underlying reference genomic coordinates. This design also allows the user to include fix patches [i.e. updates that correct errors or add sequence associated with gaps in the reference sequence; ( Schneider *et al.*, 2017)] in the graph or to recreate custom sequence using their haplotype of interest.
2- Each haplotype and nested haplotype are defined as a unique segment based on the reference genome or the closest haplotype; therefore, the number of bases that need to be stored for each haplotype sequence is minimalized.
3- Each haplotype can be uniquely represented using GFA-like notation that can track back into the node storing specific sequence for each haplotype.

**Figure 4.**
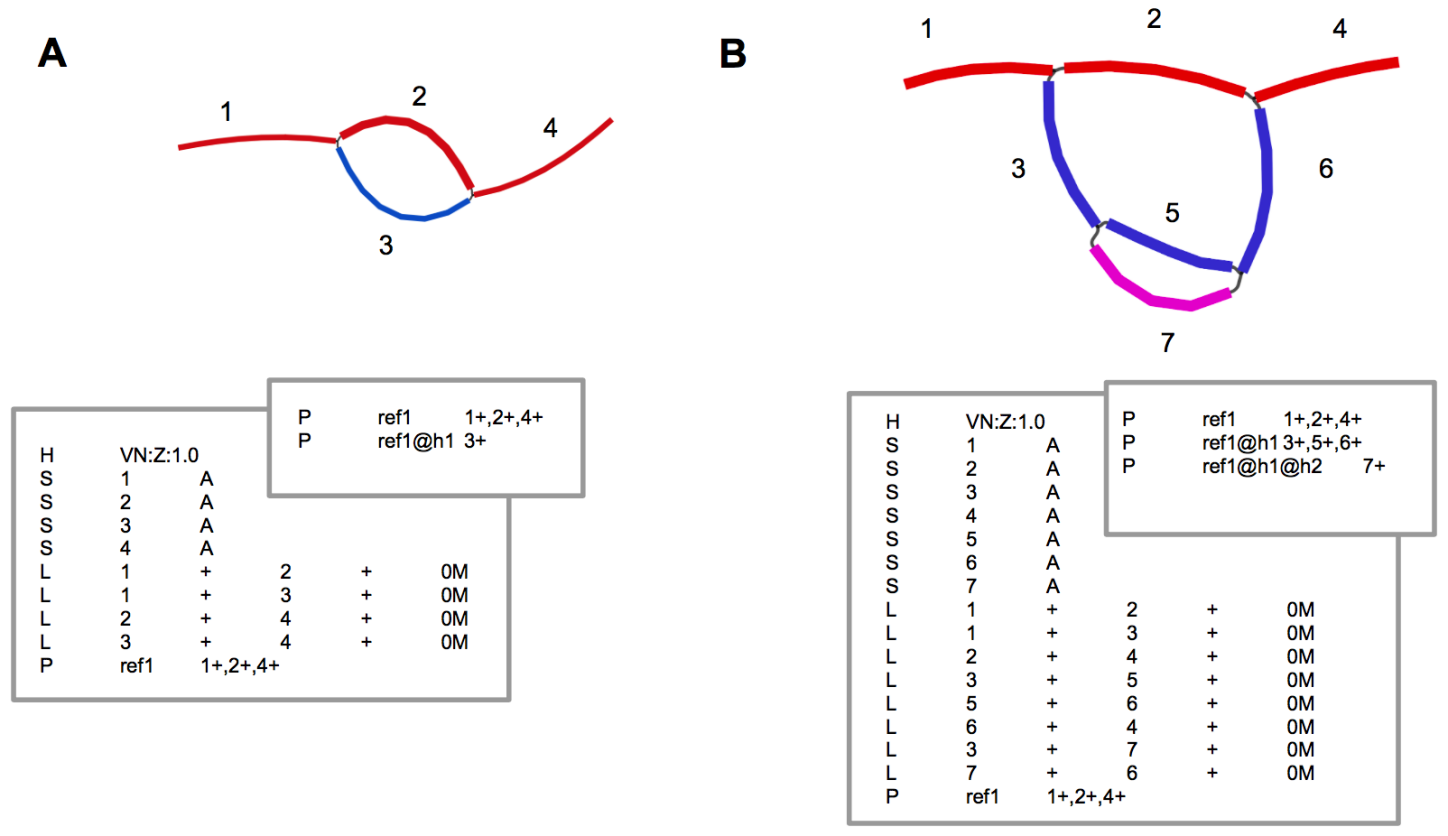
Adding additional haplotype from **A** to **B**. The existing sequence and coordinates remain the same even though the nodes and edges change.

Our proposed model allows nodes and edges represented in the GFA to change without changing the sequence corresponding to each haplotype ( Figure 4). Such an approach will be essential for future methods to both manipulate graphs that have already been constructed, as well as do comparative analyses between graphs using a common coordinate system as methods improve.

### Use case: What about plants?

The potential for applying pangenomic methods to analyze plant genomes is immense. Several new plant genomes have recently been sequenced and built upon the previously produced model plant assemblies, providing a foundation for research and end-use applications in agriculture. Crop plants form the foundation for the world's natural food and textile resources, and plant breeding efforts are often focused on improving several quality traits. A graph-based sequence-centric view of genomes sets the stage for facilitating key decisions that can be made to improve crop infrastructure.

Diversity in plants comprises an array of genome types with regard to species identity, genome size, chromosome number, and ploidy level. Pangenome studies have commenced for many of the model plant species, such as *Arabidopsis thaliana* (flowering plants) ( Clark *et al.*, 2007), *Medicago truncatula* (legumes) ( Miller *et al.*, 2017; Zhou *et al.*, 2017), and *Brachypodium distachyon* (grasses) ( Gordon *et al.*, 2017), due to the attractive attributes of their small genomes and short generation-times. Likewise, pangenome studies now target on their corresponding larger cousins, which include crop plants of economic importance such as crucifers, soybean, and wheat ( Montenegro *et al.*, 2017). These pangenome studies used highly developed sequence analyses, but not a graph-based approach. Several pangenome-related papers appear to be in preparation for other important plant species (e.g., maize); whether they all use graph-based methods remain to be seen. The exercise of testing graph-based sequence views will help formulate use-case scenarios.

Many challenges exist of course in terms of applications of graph representations of plant genomes, mostly due to their inherent complexity. One challenge is working with highly-divergent sequences during the construction of the pangenome, given the tradeoff between computational expediency and accuracy. Taking into account the transposons within plant genomes (e.g., maize as discussed above), methods relying upon global sequence alignment for whole genomes would need to address the issues of large translocations and inversions between chromosomes. Plants are often not only diploid as well, as opposed to the human genome. In sum, many pangenomic methods have had some success for verterbrate genomes (as detailed in this paper), but it is unclear how applicable these methods will be for highly complex plant genomes.

Immediate uses for graphs of plant genomes would be to validate hypothetical evolutionary tree diagrams assigned to species, and perhaps address instances where species are proposed to be ancient polyploids, or to gauge genome changes in current polyploid genomes. RNA-Seq methods may also be matched against graph-based maps to quantify expression from the genomes. For instance, it would be interesting to assess whether nutritional- or medicinal-related trait changes can be tracked to genomic structural variation using graph-based methods targeted on key metabolic pathway-associated genes. The tracking of highly repetitive transposon-initiated events may also explain some of the alterations observed in different genome species and their evolutionary consequence resulting in gene duplication, rearrangements, and the like. Use of graph-based methods to map out highly variable regions may also provide strategies toward implementing targeted engineering of species, or assist in classic breeding strategies where known attributes are known to structurally exist. Similarly, many wild ancestor lines are sought to bring in new gene function to serve as sources for disease resistance, quality traits, and nutrition; their inclusion in the graph will enable an understanding of their contributions on the whole genome scale. The construction of pangenomes by graph-based methods, and the subsequent visualization of these graphs therefore appear likely to have a valuable role in the future of agricultural improvements.

### Use case: RNA-seq mapping

Within the realm of RNA-seq, graphs can also be used to validate and benchmark analytical methods. For example, we created a spliced variation graph of chr21 using the *rna* submodule in vg (see WDL pipeline for more details) to test the RNA-seq mapping performance of vg. We used variants from the NA12878 individual in the 1000 Genomes Project ( 1000 Genomes Project Consortium *et al.*, 2015) and transcripts from the GENCODE v29 annotation ( Frankish *et al.*, 2019). The paired-end RNA-seq reads were simulated using RSEM ( Li & Dewey, 2011) from the haplotype-specific transcripts generated from *vg rna*. vg’s two mapping algorithms *map* and *mpmap* were able to align 71.6% and 73.8% of the simulated reads with a mapping quality of at least 30, respectively. This is similar to the value observed for Hisat2 using the same data. We also tested both algorithms on graphs only consisting of exonic sequences. Using these graphs, the performance increased slightly (1.5 to 2%). Due to a lack of time we were not able to finish the second part of the pipeline that involved estimating allele-specific expression from the mapped reads.

This is very much a work in progress, and work so far has only been a proof of principle. For example, all splice-junctions and variations present in the reads were also present in the graph. In addition, due to time constraints we only used the number of mapped reads as a proxy of performance and did not assess whether the reads were correctly mapped. These issues will need to be addressed in future benchmarks in order to get a more accurate estimate of vg performance on spliced variation graphs and applications for RNAseq in general. 

### Use case: Producing a fully phased diploid assembly of the HG002 MHC region

The MHC, located on human chr6, is a region highly enriched for genes and variation, including the human leukocyte antigen (HLA) which is involved in immune system function. Genetic associations between variants in this region involve different diseases, including autoimmune diseases. MHC haplotypes differ substantially, making it challenging to map reads from this region and call variants with conventional methods on a linear reference. We sought to generate a base-level accurate, fully phased, diploid assembly of the MHC of GIAB HG002 (NA24385, Ashkenazi son). The only previous studies producing fully phased, contiguous diploid assemblies for the MHC involved the NA12878 genome with PacBio reads (non-CCS) ( Jain *et al.*, 2018; Koren *et al.*, 2018). In this work, we use newer PacBio CCS and ultralong Oxford Nanopore reads, along with 10x Genomics linked-reads, to produce and carefully evaluate a targeted MHC diploid assembly for a second individual from GIAB.

The data for this work relied on sequencing results from three different PacBio CCS libraries with average read lengths of 9 kb and 13 kb for the Sequel I chemistry and 11 kb for the Sequel II chemistry, and each dataset having ~25 to 30X coverage. We also used “ultralong” data from Oxford Nanopore Technology (ONT) with total coverage of 16X (4X coverage by reads > 100 kb), and Promethion ONT data with total coverage of ~40X (~6X coverage by reads > 100 kb). We also used 10x Genomics data for phasing. HG002 reads were extracted from the MHC (HLA1/HLA2) region on GRCh37/hg19 chr6:28,477,797-33,448,354. Illumina data for the Ashkenazi father (HG003, NA24149) and mother (HG004, NA24143) from this trio was also used to bin the CCS reads by haplotype. The HLA typing reports for HG002/HG003/HG004 were generated at Stanford Blood Center on December 16, 2016.

The first data processing step involved finding reads from each haplotype mapped to MHC regions. An initial inspection of the HG002 MHC region occurred on the whole-genome de novo assembly of trio binned reads produced using the CCS data. The MHC region initially appeared to be well-assembled, with 1 contig derived from the father and 2 contigs derived from the mother, but further inspection revealed that the results were not coherent and that some of the haplotypes may possibly have been compressed. A second approach used 15 kb PacBio CCS reads that were mapped to the MHC and then selected for each haplotype. A local de novo assembly of these reads resulted in 10-15 contigs with many gaps between, although the assembly was close to the full length of the MHC. PacBio CCS reads were processed with Whatshap v0.19 to generate a phased VCF, which was then used to partition CCS reads by haplotypes. Reads for each haplotype were assembled independently into contigs that were then aligned to 10x Genomics s linked-read GemCode WGS contigs (whereby contigs were assembled with Supernova) to generate scaffolded CCS contigs for the diploid assembly. This diploid assembly was then used as the input for vg to build a genome graph via all versus all alignment (by Minimap2) followed by seqwish. 

### Confirmation of the two haplotypes

The CCS and ONT long reads were aligned to the genome graph to confirm the diploid assembly using the PedMEC phasing pipeline ( Garg *et al.*, 2016). In addition, phasing of HLA typing results in the diploid MHC assembly were also checked against the independent HLA typing results from Stanford Typing Lab, based on the proband phased haplotypes derived from the typing results of the parents (HG003 and HG004), as shown in Table 1 (the parents’ typings are not phased).

**Table 1.** Genotyping results for proband HG002 and parents HG003 and HG004.

	Proband		Father		Mother	
HLA	HG002	HG002	HG003	HG003	HG004	HG004
**A**	*26:01:01:01	*01:01:01:01	*30:01:01	*26:01:01:01	*01:01:01:01	*33:01:01
**B**	*38:01:01	*35:08:01	*13:02:01	*38:01:01	*35:08:01	*14:02:01:01
**Bw**	4	6	4		6	
**Cw**	*12:03:01:01	*04:01:01:06	*06:02:01:01	*12:03:01:01	*04:01:01:06	*08:02:01:01
**DRB1 (DR)**	*04:02:01	*10:01:01	*07:01:01:01	*04:02:01	*04:04:01	*10:01:01
**DQB1 (DQ)**	*03:02:01	*05:01:01:02	*02:02:01:01	*03:02:01	*04:02:01	*05:01:01:02
**DQA1**	*03:01:01	*01:05:01	*02:01	*03:01:01	*01:05:01	*03:03:01
**DRB3,4,5 (DR)**	4*01:03:01:01		4*01:03:01:01		4*01:03:01:01	
**DPA1 (DP)**	*01:03:01:04	*01:03:01:02	*01:03:01:04	*01:03:01:05	*01:03:01:02	*01:03:01:04
**DPB1 (DP)**	*04:01:01:01	*X	*04:01:01:01	*04:02:01:02	*04:01:01:01	*X

We will continue to explore ways graph-based analyses could be used to benchmark methods used to characterize the MHC. It will be important to identify if these haplotypes can be represented in standard VCF files with respect to the primary GRCh37/38 references in GIAB benchmark sets, or whether existing benchmarks will need new representations and benchmarking tools. Although vg can project haplotypes into a VCF file with respect to the primary reference, it remains to be determined whether this is compatible with current benchmarking tools for small variants and structural variants. Other future work will entail examining whether fully phased diploid assembly is possible in other more complex, yet medically important regions, such as those of the killer-cell immunoglobulin receptor and spinal muscular atrophy.

## Conclusion

Ongoing improvements in sequencing technology and diminishing costs make the generation of high-quality genome assemblies from diverse populations possible in a way today that could only have been imagined during the Human Genome Project (HGP). These new data are likely to form the basis for a new pangenome representation for the reference assembly that includes a graph, but they also raise many as-yet unanswered questions. We must consider the sample content, data/file formats that will be used, graph construction algorithms, how relevant metadata about quality and content will be communicated to users, and whether and how changes will be managed and tracked. New tools and validation sets must be built and community education will be essential, as will long-term curation, as is currently performed by the Genome Reference Consortium for the HGP reference. Ensuring the reference assembly remains a FAIR resource ( Wilkinson *et al*., 2016), accessible to users world-wide is also critical, and for the first time, some ethical and privacy concerns around the reference may need to be addressed. The new software developed here provide a preview of the use cases and potential for a new pangenome reference and play an important role in developing answers to these many questions. 

Gratifyingly, since this first pangenomics hackathon took place a great deal of work in the domain has been started. For example, the Human Pangenome Reference Consortium (HPRC; https://humanpangenome.org/) has been initiated by the National Human Genome Research Institute. The HPRC aims to create an updated human reference genome structure—a pangenome—good enough to replace the existing human reference, GRCh38, as a basis that will alleviate bias and so much more equally represent all of humanity. Through audacious efforts like this and other global initiatives, much work is taking place to: (i) create high-quality, reference quality genomes of a diversity of humans, (ii) organize these individuals genomes within a pangenome, (iii) develop the essential tooling that can utilize this information, and (iv) deliver compelling applications. The pipelines and tooling described in this paper represents starting points for much of this future work, and were started at the hackathon meeting.

## Data availability

### Underlying data

All data underlying the results are available as part of the article and no additional source data are required.

### Extended data

Open Science Framework: The Human Pangenome. https://doi.org/10.17605/OSF.IO/24K9N ( Busby & Biederstedt, 2019). 

Folder ‘images’, contained within folder ‘Giraffe’ contains odgi.png (Supplemental Figure 1). This file is an odgi visualization of the *Zea mays* chr10 minimap2/seqwish graph for two species. The pink and purple bars at the top represent regions of the linearized graph that are visited by each species’ chromosome path. The black lines forming an impenetrable morass below the bars represent adjacencies between graph nodes. This graph has pathologically high connectivity.

This file is available under the MIT license.

## Software availability

**For graph building and observing the GRCh38 path through a primate graph, source code and directions can be found here:** https://github.com/NCBI-Hackathons/TheHumanPangenome/tree/master/DS

**For ultra-fast read mapping to graph structures, source code and directions can be found here:** https://github.com/NCBI-Hackathons/TheHumanPangenome/tree/master/Giraffe

**Code for converting from gff3 annotations to graph annotations can be found here:** https://github.com/NCBI-Hackathons/TheHumanPangenome/tree/master/annotation

**WDL pipeline for mapping of RNA-seq data to spliced variant graphs can be found here:**

https://github.com/NCBI-Hackathons/TheHumanPangenome/tree/master/RNA

**Code for assessing structural variants with graphs can be found here:**

https://github.com/NCBI-Hackathons/TheHumanPangenome/tree/master/SV

**Code used to graph the MHC region can be found here:**

https://github.com/NCBI-Hackathons/TheHumanPangenome/tree/master/MHC

**Archived source code is available at:**https://doi.org/10.17605/OSF.IO/24K9N ( Busby & Biederstedt, 2019). 

**License:**MIT License.
