# Supplementary material for: A strategy for building and using a human reference pangenome
Source: F1000Res. 2021 Jul 29;8:1751. Originally published 2019 Oct 14. [Version 2] doi: 10.12688/f1000research.19630.2 (PMC8350888; doi:10.12688/f1000research.19630.2)
Supplement: Peer review history

# Peer review: Reviewer response for version 2

**DOI:** 10.5256/f1000research.58901.r90709

**Published:** 2021-08-13

**Authors:** Anna Kuosmanen

**Affiliations:** 1 University of Helsinki, Helsinki, Finland

No further comments to make.

Are the conclusions about the tool and its performance adequately supported by the findings presented in the article?

Yes

Is the rationale for developing the new software tool clearly explained?

Yes

Is the description of the software tool technically sound?

Yes

Are sufficient details of the code, methods and analysis (if applicable) provided to allow replication of the software development and its use by others?

Yes

Is sufficient information provided to allow interpretation of the expected output datasets and any results generated using the tool?

Yes

Reviewer Expertise:

Bioinformatics, method development

I confirm that I have read this submission and believe that I have an appropriate level of expertise to confirm that it is of an acceptable scientific standard.

# Peer review: Reviewer response for version 2

**DOI:** 10.5256/f1000research.58901.r90710

**Published:** 2021-08-06

**Authors:** Robert A. Beagrie

**Affiliations:** 1 MRC Molecular Haematology Unit, MRC Weatherall Institute of Molecular Medicine, Radcliffe Department of Medicine, University of Oxford, Oxford, UK

The authors have clarified the motivation behind the methods chosen and the conclusions they were able to draw in this revised text. The article is a useful summary of the results of the hackathon and will hopefully serve as a useful record for the community.

Are the conclusions about the tool and its performance adequately supported by the findings presented in the article?

Partly

Is the rationale for developing the new software tool clearly explained?

Yes

Is the description of the software tool technically sound?

Yes

Are sufficient details of the code, methods and analysis (if applicable) provided to allow replication of the software development and its use by others?

Yes

Is sufficient information provided to allow interpretation of the expected output datasets and any results generated using the tool?

Partly

Reviewer Expertise:

Genomics, epigenetics, gene regulation, human genetics, sequence variation.

I confirm that I have read this submission and believe that I have an appropriate level of expertise to confirm that it is of an acceptable scientific standard.

# Peer review: Reviewer response for version 1

**DOI:** 10.5256/f1000research.21527.r55209

**Published:** 2019-11-12

**Authors:** Robert A. Beagrie

**Affiliations:** 1 MRC Molecular Haematology Unit, MRC Weatherall Institute of Molecular Medicine, Radcliffe Department of Medicine, University of Oxford, Oxford, UK

Llamas, Narzisi, Schneider *et al*. present the results of a pangenomics codeathon held at UCSC this March. Specifically, they detail their progress towards creating a useable human pangenome and a set of fast and reliable software tools for manipulating and working with pangenome graphs. They compare three different methods for building a pangenome graph (Minimap/Seqwish, Cactus and Sibeliaz), suggest a prototype graph coordinates system to facilitate comparison/conversion to linear reference genomes, improve short-read mapping by vg, evaluate their graph genome performance in various use cases and provide tools for annotating pangenome graphs. Useable graph genomes that incorporate known human genetic diversity would be an incredibly useful resource for a wide range of fields, so the work presented certainly should be of broad interest. Overall, the authors have made good progress on a number of fronts, especially given the limited time available during a codeathon, however I think they could do a better job of justifying their design choices, summarising their findings and outlining necessary future steps.

 The manuscript starts by comparing three methods for building pangenome graphs to “explore the potential limitations and advantages of each method”. I fully agree that determining the best currently available method is an important first step towards a human pangenome and progress has been made towards this goal. However, the SibeliaZ part of the pipeline built a graph using a different chromosome from the other two, which will make future comparisons much more complicated. The authors do not reach the stage where they can draw conclusions about the limitations or advantages of the different methods, but at a minimum, they should outline the future steps that would need to be taken to decide on a “best” method.

 The authors propose a new graph co-ordinate system as an extension of the GFA file format, where the major difference seems to be an additional file listing the alternative haplotypes. I do not fully understand the explanation of why the new format is an improvement over GFA. The idea seems to be to allow haplotypes to be updated “separately”, yet in Figure 4 both the GFA file and the additional haplotype file need to be altered to add a new haplotype, so what is the advantage of the separate haplotype file? Whilst the coordinates of the reference do not change from Fig 4a to Fig 4b, the coordinates of haplotype 1 do. Would it not be important to maintain co-ordinates for all previously defined haplotypes when adding in new variants?

 In summary, the selection and application of software tools and methodological approaches is scientifically sound, the questions addressed are important and interesting and the manuscript does a great job of explaining the potential benefits of a pangenome graph representation over traditional linear genomes. However, the manuscript needs some rewriting to clearly articulate what the authors have learned about best practices for constructing pangenomic graphs and what they see as the next important steps on the path to constructing a high-quality human pangenome.

Are the conclusions about the tool and its performance adequately supported by the findings presented in the article?

Partly

Is the rationale for developing the new software tool clearly explained?

Yes

Is the description of the software tool technically sound?

Yes

Are sufficient details of the code, methods and analysis (if applicable) provided to allow replication of the software development and its use by others?

Yes

Is sufficient information provided to allow interpretation of the expected output datasets and any results generated using the tool?

Partly

Reviewer Expertise:

Genomics, epigenetics, gene regulation, human genetics, sequence variation.

I confirm that I have read this submission and believe that I have an appropriate level of expertise to confirm that it is of an acceptable scientific standard, however I have significant reservations, as outlined above.

# Peer review: Reviewer response for version 1

**DOI:** 10.5256/f1000research.21527.r55187

**Published:** 2019-10-29

**Authors:** Anna Kuosmanen

**Affiliations:** 1 University of Helsinki, Helsinki, Finland

The article describes the results of the first pangenomics codeathon. The purpose of the codeathon was two-fold, to propose technical specifications and standards for a usable human pangenome and to build tools for genome graphs.

 The traditional representation of a reference genome is a set of linear sequences (chromosomes), with possibly additional alternative sequences to capture variations. An alternative to a linear reference genome is a "pangenome", a representation of all genomic variation observed in a population. Pangenomes are modeled as graphs in this article. The article discusses the benefits of a pangenome reference over the traditional reference, and describes several software tools and pipelines for pangenomics applications.

 The authors explain very well the limitations of the linear reference genome, and describe how a pangenome graph reference would be superior. And it is great that the Conclusions section also raises important non-technical matters related to pangenomes, such as privacy concerns.

 The tools and pipelines described in the article build on VG, with some of them being very much work in progress, as is natural for the results of a codeathon. All the tools and pipelines, as well as the data used in the codeathon, are described in detail, and additionally all the code is available on github, allowing for easy replication of the development. Github also has detailed instructions on the use of the tools/pipelines and examples of the output.

 The article organization is at times confusing. The methods section consists of two parts: Implementation and Use cases, with the topics of Use cases and Implementation overlapping. But each category also has topics which are not in the other. The distinction between these two categories isn't clear either, as depending on the topic the data and/or methods descriptions can be found in one or the other (e.g. in "graph coordinate system", data and methods are described in Implementation, and "RNA-seq mapping" has all the data and methods in "Use cases").

 In my opinion the article is scientifically sound, but it could use some re-structuring for better readability.

 Minor comments:

1. For building the graphs you describe the first two methods in detail, but for third you simply say "In addition, we used SibeliaZ to build a graph...". The third method could use a sentence or two about it too.
2. Figure 1: I found this slightly confusing that there's a GFA file that has one path ("P") line, and then there's "a path file accompanying the GFA file" with two path lines (of which one is in the former).
3. In section "Pipeline for mapper evaluation on maize graphs", it is unclear what is the goal of this experiment till you look at Figure 3. The first sentence of the section also sounds odd, like there are words missing ("We also sought to test a plant model..."), and the wording of "comparing graphs" is in conflict with Figure 3 text.
4. Typo in GSNAP ("GNSAP") in section "Mapping RNA sequencing data to variant graphs".
5. In section "Use case: Producing a fully phased diploid assembly of the HG002 MHC region", what kind of data is 10X Genomics data?

Are the conclusions about the tool and its performance adequately supported by the findings presented in the article?

Yes

Is the rationale for developing the new software tool clearly explained?

Yes

Is the description of the software tool technically sound?

Yes

Are sufficient details of the code, methods and analysis (if applicable) provided to allow replication of the software development and its use by others?

Yes

Is sufficient information provided to allow interpretation of the expected output datasets and any results generated using the tool?

Yes

Reviewer Expertise:

Bioinformatics, method development

I confirm that I have read this submission and believe that I have an appropriate level of expertise to confirm that it is of an acceptable scientific standard.
